# Spatiotemporal characteristic analysis of PM_2.5_ in central China and modeling of driving factors based on MGWR: a case study of Henan Province

**DOI:** 10.3389/fpubh.2023.1295468

**Published:** 2023-12-05

**Authors:** Hua Wang, Mingcheng Zhang, Jiqiang Niu, Xiaoyun Zheng

**Affiliations:** ^1^School of Computer and Communication Engineering, Zhengzhou University of Light Industry, Zhengzhou, China; ^2^Key Laboratory for Synergistic Prevention of Water and Soil Environmental Pollution, Xinyang Normal University, Xinyang, China; ^3^Key Laboratory of Urban Land Resources Monitoring and Simulation, Ministry of Natural Resources, Shenzhen, China

**Keywords:** PM_2.5_, spatiotemporal variation, MGWR, Central China, air quality

## Abstract

Since the start of the twenty-first century, China's economy has grown at a high or moderate rate, and air pollution has become increasingly severe. The study was conducted using data from remote sensing observations between 1998 and 2019, employing the standard deviation ellipse model and spatial autocorrelation analysis, to explore the spatiotemporal distribution characteristics of PM_2.5_ in Henan Province. Additionally, a multiscale geographically weighted regression model (MGWR) was applied to explore the impact of 12 driving factors (e.g., mean surface temperature and CO_2_ emissions) on PM_2.5_ concentration. The research revealed that (1) Over a period of 22 years, the yearly mean PM_2.5_ concentrations in Henan Province demonstrated a trend resembling the shape of the letter “M”, and the general trend observed in Henan Province demonstrated that the spatial center of gravity of PM_2.5_ concentrations shifted toward the north. (2) Distinct spatial clustering patterns of PM_2.5_ were observed in Henan Province, with the northern region showing a primary concentration of spatial hot spots, while the western and southern areas were predominantly characterized as cold spots. (3) MGWR is more effective than GWR in unveiling the spatial heterogeneity of influencing factors at various scales, thereby making it a more appropriate approach for investigating the driving mechanisms behind PM_2.5_ concentration. (4) The results acquired from the MGWR model indicate that there are varying degrees of spatial heterogeneity in the effects of various factors on PM_2.5_ concentration. To summarize the above conclusions, the management of the atmospheric environment in Henan Province still has a long way to go, and the formulation of relevant policies should be adapted to local conditions, taking into account the spatial scale effect of the impact of different influencing factors on PM_2.5_.

## 1 Introduction

China's economy has expanded significantly since the reform and opening up, but the quality of the environment has deteriorated (1), and atmospheric environmental pollution has become the focus of society and one of the most urgent problems to be solved by governments at all levels (2). PM_2.5_ is a major atmospheric pollutant (3) and is also known as fine particulate matter and fine particles; PM_2.5_ refers to atmospheric airborne particles with a diameter of 2.5 microns or smaller. Studies have shown that it seriously harms the atmospheric environment and human health (4–6).

Presently, more academics domestically and internationally have conducted investigations of PM_2.5_ geographical and temporal distribution patterns and related factors at various spatial scales, and a multiscale research system has been formed for national (7–9), river basin (10–12), city cluster (13–15), provincial (16–21), and prefecture-level city scales (22–24). Studies based on the provincial scale have focused mostly on Beijing-Tianjin-Hebei and the relatively economically developed regions in the south. However, there are relatively few studies on the spatiotemporal distribution characteristics and driving mechanisms of PM_2.5_ in various provinces in Central China (25–27). On April 15, 2006, the “Several Opinions of the CPC Central Committee and The State Council on Promoting the Rise of the Central Region” was officially promulgated. Central China is an important bearing area for China's “Rise of Central China” strategy and an important area for the implementation of coordinated regional development and new industrialization strategy. The sustained economic development of China as a whole is strategically dependent on the region's economic success. Central China's Henan Province is a significant economic, populated, and recently industrialized region, and the 2019 China Ecological Environment Status Bulletin published by the Ministry of Ecology and Environment showed that from January to December 2019, six of the twenty cities with relatively poor air quality among 168 key cities in China, including Anyang and Jiaozuo, were located in Henan Province, and the majority of Henan Province was located in the high-concentration area of PM_2.5_ pollution, which is the Yellow Huaihai Plain (28). Studying the spatiotemporal pattern and driving mechanism of PM_2.5_ in Henan Province is of great significance for understanding the spatiotemporal distribution properties of PM_2.5_ in Henan Province, promoting sustainable development, and safeguarding the health of the population.

The goal of pertinent study should be to investigate the spatiotemporal distribution properties of PM_2.5_ and the underlying causes of its existence, according to present research findings. From the studies of the spatiotemporal distribution characteristics of PM_2.5_, scholars have mainly explored PM_2.5_ spatiotemporal variation patterns and spatial aggregation characteristics through spatial center of gravity shift evaluation, cold and hot spot analysis, and spatial autocorrelation analysis (29–33). Among them, the spatial center of gravity shift can be realized by the standard deviation ellipsoid (SDE) model (34, 35), which can not only calculate the center of gravity of the PM_2.5_ concentration spatial distribution but also effectively reflect the spatial aggregation trend of PM_2.5_. Regarding the investigation of PM_2.5_ driving mechanisms, the driving mechanism research methods that have been applied can be divided into traditional research methods such as Pearson correlation coefficient analysis (36), rank correlation analysis (37), gray correlation analysis (38), and OLS regression models, as well as methods that introduce a spatial perspective such as the geographical detector method (39, 40), spatial lag model (41), spatial error model (42), spatial Durbin model (43), and geographically weighted regression model (GWR) (44). It has been shown that PM_2.5_ concentration, as a kind of data with spatial properties, has a certain degree of spatial heterogeneity for each influencing factor (45). It has been found that the interaction between indicators with spatial attributes, such as PM_2.5_ concentration, and their associated influencing factors tends to have multiscale effects (46, 47). To a certain degree, GWR, functioning as a local regression model, is capable of depicting the spatial heterogeneity of driving factors affecting PM_2.5_. The MGWR method proposed by Fotheringham in 2017 provides a new way (48) to describe the spatial heterogeneity of the influence of independent variable. In addition, MGWR introduces the idea of a multiscale perspective, which is a very important issue in geography (49). Therefore, comparing the GWR with the MGWR and selecting the better-fitting model to analyze the driving mechanism is a more reliable research method to obtain an accurate PM_2.5_ concentration driving model.

In summary, this paper intends to take Henan Province as the study area, based on the PM_2.5_ remote sensing data from 1998 to 2019, which have been corrected and fused, as well as high spatial resolution factor raster data. The study analyzed the spatiotemporal distribution characteristics of PM_2.5_ using the standard deviation ellipse model and explored the spatial aggregation characteristics of PM_2.5_ concentration in Henan Province through methods such as spatial autocorrelation analysis and hot and cold spot analysis. Furthermore, this paper employs the MGWR model in combination to disclose how driving factors impact the distribution pattern and spatial heterogeneity of PM_2.5_, providing a reference for analyzing the pollution situation of PM_2.5_ and creating regulations for the atmospheric environment's sustainable development.

## 2 Data and methods

### 2.1 Study area overview

**Figure 1:**
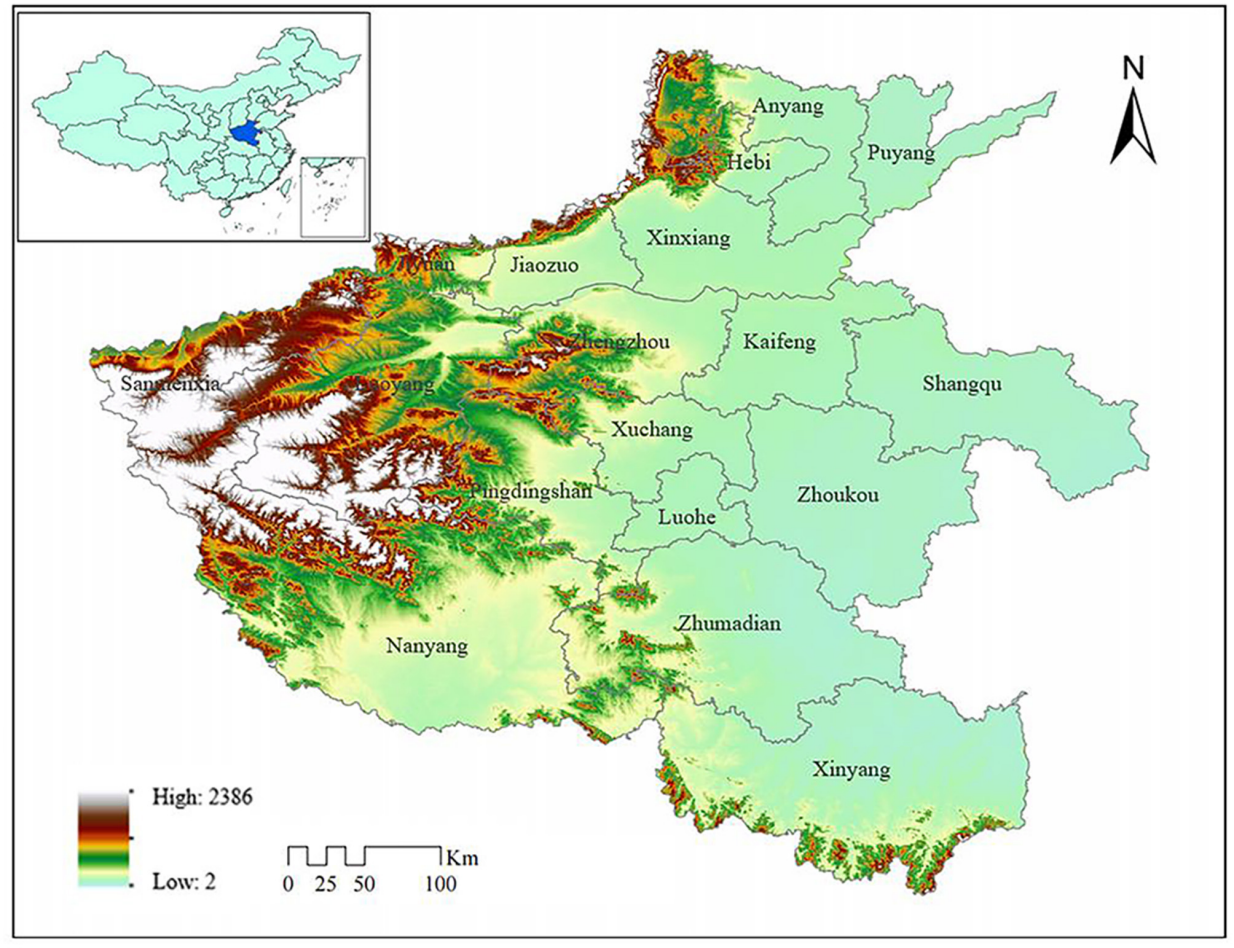
The geographical position and administrative divisions of Henan Province.

Henan Province is located in the central hinterland of China (Figure 1), with complex terrain and rich resources. Henan Province is of great importance as a key grain production region, an energy and raw material base, and a comprehensive transportation hub in China. With abundant human resources and scientific and educational resources, the province is an important agricultural province and a populous province in China. In addition, with the proposal of the “rise of Central China” strategy, the industrialization development of Henan Province also ushered in the spring, and it has now become a large emerging industrial province, making progress in economic development. Henan Province also has serious air quality problems, according to the “Annual Work Program for Air Pollution Prevention and Control in Henan Province (2019-2020)” released in 2019; it was stated that the yearly mean PM_2.5_ concentration in Henan Province was 53 μg/m^3^, up 3.9% year-on-year, the ratio of good days was 55.8%, down 2.5% year-on-year, and the management of atmospheric environment needs to be improved.

### 2.2 Study data

#### 2.2.1 PM_2.5_ data

The PM_2.5_ remote sensing data utilized in this study were derived from the Atmospheric Composition Analysis Group at Washington University (St. Louis) (https://sites.wustl.edu/acag/datasets/surface-pm2-5). The data span from 1998 to 2019 with a spatial resolution of ~1 × 1 km, and the product number for the Chinese region is V4.CH.03. With its great spatial resolution, the data product can depict the spatial distribution pattern of PM_2.5_; additionally, the product has been widely used in global and regional PM_2.5_-related studies (50–52).

#### 2.2.2 Other data

To unpack the driving mechanism of PM_2.5_ spatial concentration, this study introduced two types of influence factor data: natural type data and socioeconomic type data. The natural-type data included surface temperature, relative humidity, wind speed, precipitation, vegetation index, and elevation. The socioeconomic data included GDP, nighttime lights, CO_2_ emissions, electricity consumption, population density, and arable land area share. Electricity consumption data were obtained from Chen et al. (53) and published in Scientific Data. Yang et al. (54) supplied a land cover dataset covering the period from 1990 to 2019 in China, with a spatial resolution of 30 m. Using this dataset, the data concerning the proportion of arable land area was computed. The specific data description and data sources are shown in Table 1.

**Table 1:** Factors and descriptions of PM_2.5_ concentration impact.

**Type**	**Factor**	**Explanation**
Natural factors	X1: surface temperature	Average annual surface temperature in Henan Province (°C) [Resource and Environmental Science and Data Center, Chinese Academy of Sciences (https://www.resdc.cn/DOI/DOI.aspx?DOIID=98)]
	X2: relative humidity	Annual average relative humidity in Henan Province (kg/kg) [National Earth System Science Data Center Data Details (http://www.geodata.cn/data/datadetails.html?dataguid=126928059243667&docId=11969)]
	X3: wind speed	Annual average wind speed in Henan Province (m/s) [National Earth System Science Data Center Data Details (http://www.geodata.cn/data/datadetails.html?dataguid=3796451&docid=5735)]
	X4: precipitation	Annual precipitation in Henan Province (0.1 mm) [National Earth System Science Data Center Data Details (http://www.geodata.cn/data/datadetails.html?dataguid=192891852410344&docid=4)]
	X5: vegetation index	Normalized vegetation index in Henan Province (https://modis.gsfc.nasa.gov/data/dataprod/mod13.php)
	X6: elevation	Elevation of Henan Province (m) [MODIS Web (https://modis.gsfc.nasa.gov/)]
Social factors	X7: GDP	GDP of Henan Province (million yuan/square kilometer) [Resource and Environmental Science and Data Center (https://www.resdc.cn/DOI/DOI.aspx?DOIID=33)]
	X8: nighttime light	Henan Province night light brightness (LUX) [National Qinghai-Tibet Plateau Scientific Data Center (https://data.tpdc.ac.cn/zh-hans/data/e755f1ba-9cd1-4e43-98ca-cd081b5a0b3e)]
	X9: CO_2_ emissions	Annual CO_2_ emissions in Henan Province (tons/square kilometer) [Center for Global Environmental Research, National Institute for Environmental Studies, Japan (https://cger.nies.go.jp/en/)]
	X10: electricity consumption	Annual electricity consumption in Henan Province (kWh) (https://www.nature.com/articles/s41597-022-01322-5)
	X11: population density	Population Density in Henan Province (persons/square kilometer) (ORNL LandScan Viewer—Oak Ridge National Laboratory) https://landscan.ornl.gov/
	X12: arable land area share	Percentage of arable land in Henan Province by county (%) [ESSD—The 30 m annual land cover dataset and its dynamics in China from 1990 to 2019 (https://essd.copernicus.org/articles/13/3907/2021/)]

After random sampling and eliminating invalid sampling data in Henan Province, 2,956 sample units of natural and socioeconomic data were obtained. Considering the possibility of multicollinearity in the variables selected in this study, the variance inflation factor (VIF) was used to test all the abovementioned explanatory variables to avoid bias in the estimation results caused by the interaction between indicators. It is generally assumed that if the VIF is >10, the variable is highly collinear. The inverse of the VIF is the tolerance, and the closer it is to 0, the stronger the multicollinearity is. The VIF is computed using the following formula.


(1) $$\begin{array}{l} VIF=\frac{1}{1-R_{i}^{2}} \end{array}$$

The value of *Ri* represents the complex correlation coefficient between the *i*th independent variable (*Xi*) and the other independent variables employed in the regression analysis. Table 2 presents the results of the test. The VIF values of each indicator are <10, declaring that the selected indicators do not have multicollinearity problems.

**Table 2:** Multicollinearity inspection of influencing factors.

**Variable**	**X1**	**X2**	**X3**	**X4**	**X5**	**X6**	**X7**	**X8**	**X9**	**X10**	**X11**	**X12**
VIF	2.683	3.850	2.742	3.080	2.210	6.136	1.719	3.003	1.017	2.664	1.214	2.667
Tolerance	0.373	0.260	0.365	0.325	0.453	0.163	0.582	0.333	0.983	0.375	0.823	0.374

### 2.3 Research methodology

#### 2.3.1 Standard deviation ellipse analysis

SDE is an algorithm that visually evaluates the orientation and distribution characteristics of a series of discrete points. In this paper, the standard deviation ellipse is used to reflect the spatiotemporal evolution pattern of PM_2.5_ in the past 22 years, mainly using the parameters of center of gravity, area, long and short axes, and azimuth Angle of the standard deviation ellipse. The ellipse center $\left({\bar{X}}_{w},{\bar{Y}}_{w}\right)$ is calculated by the following formulas:


(2) $$\begin{array}{c} {\bar{X}}_{w}=\frac{\overset{n}{\underset{i=1}{\sum }}w_{i}x_{i}}{\overset{n}{\underset{i=1}{\sum }}w_{i}} \end{array}$$

(3) $$\begin{array}{c} {Ȳ}_{w}=\frac{\overset{n}{\underset{i=1}{\sum }}w_{i}y_{i}}{\overset{n}{\underset{i=1}{\sum }}w_{i}} \end{array}$$

where *w*_*i*_ is the weight of object *i* and (*x*_*i*_, *y*_*i*_) is the spatial coordinate location of study object *i*.

The azimuth angle, which is the angle of clockwise rotation from due north to the long axis direction, reflects the primary trend direction of the PM_2.5_ data distribution. The change in azimuth can be a reflection of the primary trend direction of the data distribution for PM_2.5_ concentrations. The azimuth angle is calculated by the following formulas:


(4) $$\begin{array}{l} \tan\theta \\ =\frac{\left(\overset{n}{\underset{i=1}{\sum }}w_{i}^{2}{\tilde{x}}_{i}^{2}-\overset{n}{\underset{i=1}{\sum }}w_{i}^{2}{\tilde{y}}_{i}^{2}\right)+\sqrt{\left(\overset{n}{\underset{i=1}{\sum }}w_{i}^{2}{\tilde{x}}_{i}^{2}-\overset{n}{\underset{i=1}{\sum }}w_{i}^{2}{\tilde{y}}_{i}^{2}\right)+4\overset{n}{\underset{i=1}{\sum }}w_{i}^{2}{\tilde{x}}_{i}^{2}{\tilde{y}}_{i}^{2}}}{2\overset{n}{\underset{i=1}{\sum }}w_{i}^{2}{\tilde{x}}_{i}{\tilde{y}}_{i}} \end{array}$$

where $\left({\tilde{x}}_{i},{ỹ}_{i}\right)$ represents the deviation of the spatial coordinates of the PM_2.5_ concentration in the study area to the center point $\left({\bar{X}}_{w},{\bar{Y}}_{w}\;\right)$.

The ellipse's long half-axis denotes the distribution's direction, while its short half-axis denotes its range for PM_2.5_ data. The data's centripetal force is more visible when the short half-axis is shorter; when it is longer, the PM_2.5_ data are more distributed. As the distance between the long and short half-axes increases, the directionality of the PM_2.5_ data becomes increasingly obvious.

The formulas for calculating the long and short semiaxes are as follows:


(5) $$\begin{array}{c} \sigma_{x}=\sqrt{\frac{\overset{n}{\underset{i=1}{\sum }}{\left(w_{i}{\tilde{x}}_{i}\cos\theta -w_{i}{\tilde{y}}_{i}\sin\theta \right)}^{2}}{\overset{n}{\underset{i=1}{\sum }}w_{i}^{2}}} \end{array}$$

(6) $$\begin{array}{c} \sigma_{y}=\sqrt{\frac{\overset{n}{\underset{i=1}{\sum }}{\left(w_{i}{\tilde{x}}_{i}\sin\theta -w_{i}{\tilde{y}}_{i}\cos\theta \right)}^{2}}{\overset{n}{\underset{i=1}{\sum }}w_{i}^{2}}} \end{array}$$

where the axes' relative standard deviations along *x* and *y* are represented by σ_*x*_ and σ_*y*_, respectively.

#### 2.3.2 Spatial autocorrelation analysis

Spatial autocorrelation analysis is a potential interdependence in geography that can be used to describe the interdependence between two points that are geographically close to each other and the interdependence between temporal variables at different locations. Atmospheric activities have a strong spatial continuity, and thus, the PM_2.5_ concentration values are closer when they are spatial close to each other. Statistics for spatial autocorrelation are frequently used to examine the spatial clustering and cyclical patterns of geographic elements and can be used to depict the interdependence between geographic observation data.


(7) $$\begin{array}{c} I=\frac{\overset{n}{\underset{i=1}{\sum }}\overset{n}{\underset{j=1}{\sum }}W_{ij}\left(X_{i}-\bar{X}\right)\left(X_{j}-\bar{X}\right)}{{\left(\frac{1}{n}\overset{n}{\underset{i=1}{\sum }}X_{i}-\bar{X}\right)}_{\;}^{2}\overset{n}{\underset{i=1}{\sum }}\overset{n}{\underset{j=1}{\sum }}W_{ij}} \end{array}$$

where *I* denotes the global Moran index, *n* is the number of observation cells, *X*_*i*_ and *X*_*j*_ are the PM_2.5_ concentrations of cells *i* and *j*, *W*_*ij*_ denotes the spatial weight between points *i* and *j*, a *W*_*ij*_ equal to 1 means they are adjacent, a *W*_*ij*_ equal to 0 means they are not adjacent, and $\bar{X}$ is the sample mean. The Moran index is between [−1, 1], When *I* > 0, it signifies positive spatial autocorrelation, which means that the observed attributes are spatially clustered. On the other hand, when *I* < 0, it indicates negative spatial autocorrelation, i.e., the observed attributes are discrete in space.

#### 2.3.3 Hot spot analysis with rendering (Getis-Ord ${\text{G}}_{\text{i}}^{*}$)

The global Moran index reflects only the overall autocorrelation of PM_2.5_. In this study, the hot spot analysis method was used to analyze the local autocorrelation of PM_2.5_. The hot spot analysis method identifies spatial clusters of statistically significant low and high values, i.e., cold and hot spots, by all values in the local area. The Getis-Ord $G_{i}^{*}$ local analysis is calculated by the following formulas:


(8) $$\begin{array}{c} G_{i}^{*}=\frac{\overset{n}{\underset{j=1}{\sum }}W_{ij}X_{j}-\bar{X}\overset{n}{\underset{j=1}{\sum }}W_{ij}}{S\sqrt{\frac{\left[n\overset{n}{\underset{j=1}{\sum }}W_{ij}^{2}-{\left(\overset{n}{\underset{j=1}{\sum }}W_{ij}\right)}_{\;}^{2}\right]}{n-1}}} \end{array}$$

(9) $$\begin{array}{c} \bar{X}=\frac{\overset{n}{\underset{j=1}{\sum }}X_{j}}{n} \end{array}$$

(10) $$\begin{array}{c} S=\sqrt{\frac{\overset{n}{\underset{j=1}{\sum }}X_{j}^{2}}{n}-{\bar{X}}^{2}} \end{array}$$

where *n* is the number of spatial data cells; *X*_*j*_ is the attribute value of data cell *j*; which indicates the spatial adjacency of cell *i* and cell *j*; *S* is the average standard deviation of PM_2.5_ for the whole research region; A high value of $G_{i}^{*}$ indicates dense clustering of hot spots, and vice versa indicates dense clustering of cold spots.

#### 2.3.4 Multiscale geographically weighted regression

MGWR is an extension and improvement of GWR (48). Before introducing MGWR, the classical OLS and traditional GWR models were first introduced in the context of PM_2.5_ research.

The classical OLS model is a global model widely used for relationship analysis with the following equation:


(11) $$\begin{array}{c} Yi=\beta_{0}+\overset{n}{\underset{j=1}{\sum }}\beta_{j}X_{ij}+\varepsilon_{i} \end{array}$$

where *i* denotes a cell, *Y*_*i*_ denotes the yearly mean PM_2.5_ mass concentration of cell *i*, *X*_*ij*_ is the *j*th explanatory variable of cell *i*, β is an unknown parameter to be estimated for the association between PM_2.5_ concentration data and covariates, and ε_*i*_ is a random error component.

The GWR model can resolve the spatial autocorrelation and spatial non-smoothness problems that cannot be solved by the OLS model. Its basic formula is as follows:


(12) $$\begin{array}{c} Yi=\beta_{0}\left(u_{i},v_{i}\right)+\overset{n}{\underset{j=1}{\sum }}\beta_{j}\left(u_{i},v_{i}\right)X_{ij}+\varepsilon_{i} \end{array}$$

where β_0_(*u*_*i*_, *v*_*i*_) for the cell *i* constant term, β_*j*_(*u*_*i*_, *v*_*i*_) for the regression coefficient of the independent variable at the data sampling point.

Based on the GWR model, MGWR loosens the assumption of “same spatial scale” and can obtain the optimal bandwidth of different variables, thus reducing the bias of estimates. The formula is as follows:


(13) $$\begin{array}{c} Yi=\beta_{bw0}\left(u_{i},v_{i}\right)+\overset{n}{\underset{j=1}{\sum }}\beta_{bwj}\left(u_{i},v_{i}\right)X_{ij}+\varepsilon_{i} \end{array}$$

The main difference between Equations (13) and (12) is that *bwj* represents the bandwidth used for the regression coefficients of the *j*th variable, and β_*bwj*_(*u*_*i*_, *v*_*i*_) is the regression coefficient of the *j*th variable at cell *i*. The MGWR model was calibrated by the reverse fitting algorithm proposed by Fotheringham et al. (48, 55).

The construction of the MGWR model in this paper was based on the development of MGWR2.2 software by the Spatial Analysis Research Center (SPARC) at Arizona State University (https://sgsup.asu.edu/SPARC).

The spatiotemporal distribution of PM_2.5_ is influenced by both natural geographical factors and social and economic constraints. Therefore, this paper comprehensively considers the scientificity and accessibility of indicators and selects 12 factors from both social and natural aspects for analysis.

## 3 Results and analysis

### 3.1 Temporal variation pattern of PM_2.5_ concentration

**Figure 2:**
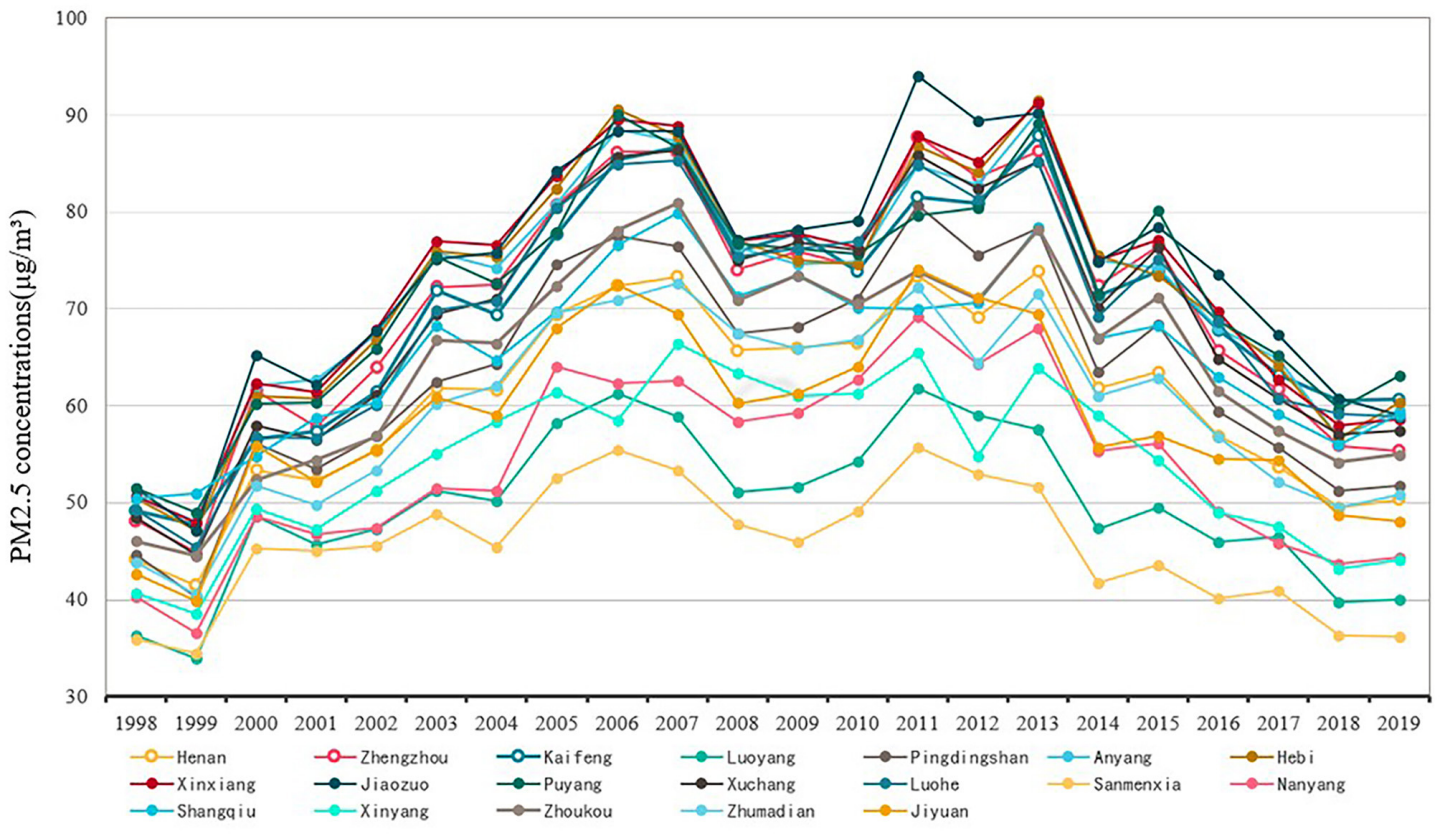
The trend of yearly mean concentration change of PM_2.5_.

Based on the remote sensing retrieval data of PM_2.5_, the average annual PM_2.5_ concentration at provincial and municipal levels during 1998–2019 was calculated., and according to the line graph drawn (Figure 2), it was observed that from 1998 to 2019, the yearly mean PM_2.5_ concentrations in Henan Province and municipalities in the province showed an “M”-shaped trend, with peaks in 2007 and 2013 at 73.35 and 73.85 μg/m^3^, respectively, which were both higher than the Ambient Air Quality Standard (GB3095-2012) of the corresponding secondary standard limit value of 35 μg/m^3^. From 1998 to 2007, the PM_2.5_ concentrations showed an overall upward trend. After a brief downturn in the early 1990s, the industry in Henan Province experienced a phase of rapid growth from 1998 onwards, which induced a yearly increase in PM_2.5_ concentrations. After a brief decline between 2007 and 2013, the PM_2.5_ quality concentrations were brought under control in approximately 2008, mainly as a result of strict emission reduction measures for the Beijing Olympics. The overall decrease in PM_2.5_ concentration from 2013 to 2019 reflects the successful implementation of The State Council's 2013 Action Plan for the Prevention and Control of Air Pollution. It is worth stating that the overall annual average PM_2.5_ concentration in Henan Province has been decreasing each year since 2015, which shows that the “Notice on Strengthening Straw Burning Ban and Comprehensive Utilization” promulgated by the General Office of Henan Provincial People's Government in 2015 has achieved certain results in straw burning ban and comprehensive utilization.

At the city scale, Jiaozuo city and Xinxiang city had the highest annual average PM_2.5_ concentration among the 18 cities, Which was connected to the energy and industrial structures in the area. Heavy industry and the energy sector, which have a bigger environmental impact, dominate the local industrial structure. Additionally, there are a lot of coal deposits in the area. The electricity demand mainly relies on thermal power generation, which also aggravates the level of PM_2.5_ pollution. The low yearly mean PM_2.5_ concentrations in Luoyang and Jiyuan are because Luoyang is situated in Henan Province's western region and has a relatively dry and cold climate, which does not encourage the buildup of PM_2.5_ contaminants. This result also shows that the “Regulations on the Ban of Fireworks in Luoyang City” promulgated by the Ecological Environment Bureau of Luoyang City in 2005 has had some beneficial environmental effects. The industrial structure of Jiyuan city is dominated by light industry, which has only a minor environmental impact.

**Figure 3:**
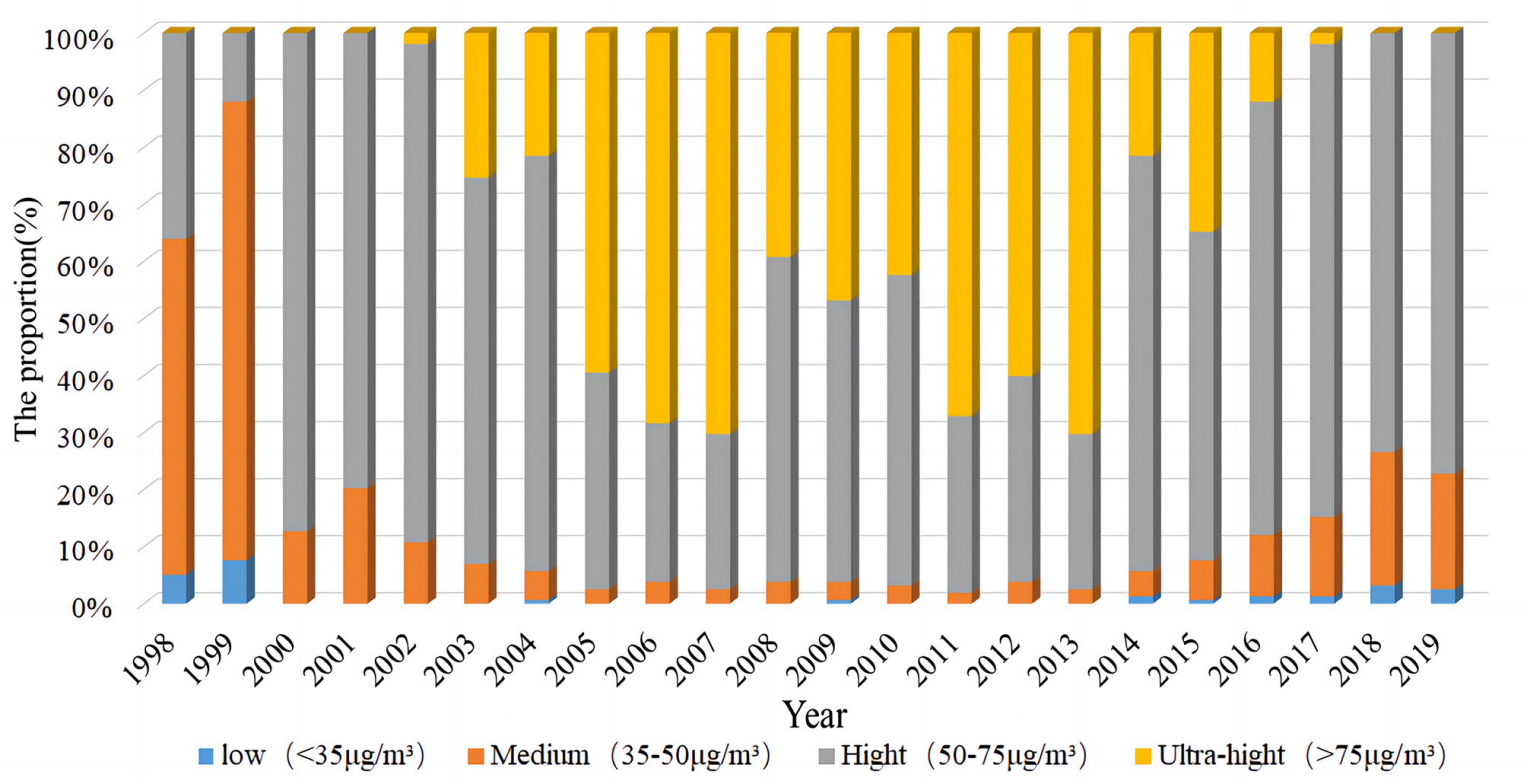
Trends of PM_2.5_ concentration changes by range in Henan provincein 1998–2019.

In order to gain insights into the temporal variation of PM_2.5_ concentration, using the yearly mean limits of PM_2.5_ concentration as outlined in the Ambient Air Quality Standard (GB3095-2012) by the Ministry of Environmental Protection of China in 2012, the study divides the yearly mean PM_2.5_ concentration into four intervals. Subsequently, the distribution of districts and counties within each interval is carefully analyzed during the study period, with a visual representation provided in Figure 3. The results show that: (1) There were few counties and districts in Henan Province with yearly average PM_2.5_ concentration levels below 35 g/m^3^. This indicates that there is no large-scale occurrence of PM_2.5_ low-value areas in Henan Province, and the overall air quality is worse. (2) From 1998 to 2019, the number of districts and counties with an yearly mean PM_2.5_ concentration values >75 μg/m^3^ first gradually increased with the development of industrialization in Henan Province, and then decreased to 0 with the government's gradual attention to PM_2.5_ pollution, which showed that PM_2.5_ pollution management in Henan Province had also achieved some results in recent years. (3) There were more districts and counties with yearly mean PM_2.5_ concentrations between 50 and 75 μg/m^3^, indicating that more districts and counties than necessary exceeded the national secondary standard and that there was still much work to be done in Henan Province to manage PM_2.5_ pollution.

### 3.2 Spatial variation trend analysis of PM_2.5_ concentration

**Figure 4:**
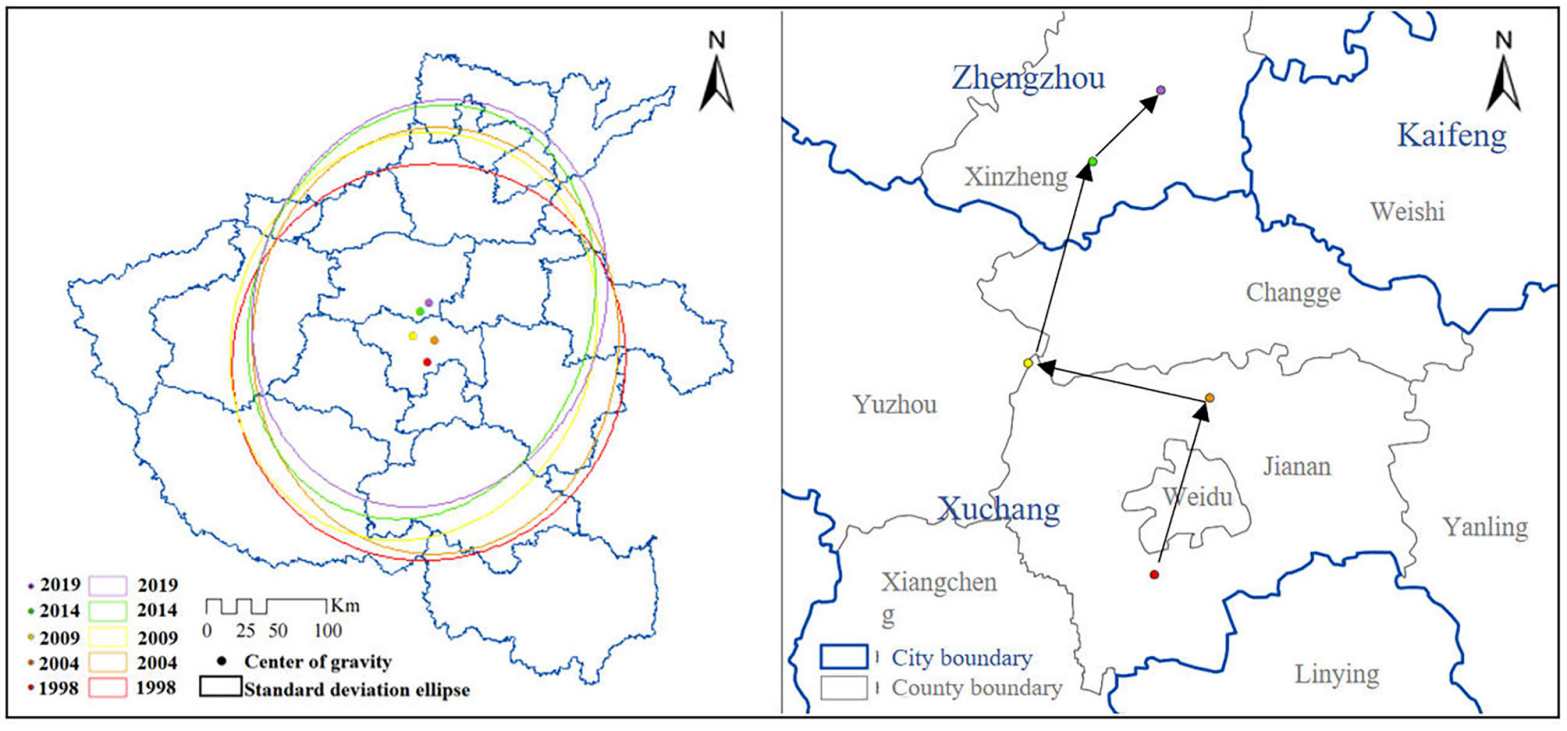
Spatial variation in the PM_2.5_ concentration center of gravity and standard deviation ellipse.

In order to deeply explore the spatiotemporal pattern of PM_2.5_ concentration, this paper used the standard deviation ellipse to quantitatively explain the spatiotemporal evolution characteristics of the average annual PM_2.5_ concentration distribution in Henan Province from a global perspective, such as the shift of the center of gravity, the contraction trend, and the direction of distribution. Only the results for 1998, 2004, 2009, 2014, and 2019 are shown here, and the details are shown in Table 3 and Figure 4.

**Table 3:** Standard deviation ellipse parameters.

**Year**	**1998**	**2004**	**2009**	**2014**	**2019**
Azimuth/°	35.84	2.76	21.05	19.12	19.01
Location of the center of gravity	Jianan	Jianan	Yuzhou	Xinzheng	Xinzheng
Ellipse area/km^2^	139,536	136,265	129,926	124,517	125,375
Long axis standard deviation/km	210.814	224.853	218.370	222.544	217.933
Short-axis standard deviation/km	205.261	192.911	189.397	178.108	183.130

In terms of azimuthal variation, the PM_2.5_ concentration in Henan Province shows a spatial distribution pattern of “northeast-southwest”, which is roughly related to the geographical landscape and population distribution in Henan Province. The azimuth angle fluctuated from 35.84° in 1998 to 19.01° in 2019, indicating that there was a weak trend of the PM_2.5_ spatial distribution pattern shifting to the “north-south” direction, which means that Jiaozuo city, Xinxiang city, and their surrounding areas, which are roughly located in the northern Henan Province, have a slightly stronger air pollution level than the northeastern part. This result is consistent with the industrial and energy structure of northern Henan Province. The standard deviation ellipse's long and short axes' differences revealed a general tendency of gradually increasing, which further demonstrated the directionality of the spatial distribution of PM_2.5_ concentrations. From the direction of the long axis, the standard deviation of the long axis increased from 210.814 km in 1998 to 224.853 km in 2004, which means that the PM_2.5_ expanded in the direction of “northeast-southwest”, i.e., the high pollution area was concentrated. From 2004 to 2009, the standard deviation of the long axis decreased from 224.853 to 218.370 km, indicating that the air pollution in this period showed a shrinking trend in the main direction. This outcome is in line with the 22-year trend of Henan Province's yearly mean PM_2.5_ concentration. Indicating that the spatial centripetal force of the PM_2.5_ concentration initially increased and then dispersed, the standard deviation of the short axis showed a tendency of first reducing and then increasing. Overall, the geographical spillover effect has been highlighted as the PM_2.5_ concentration in Henan Province tends to expand and scatter in geographic areas.

In terms of the spatial center of gravity, the center of gravity of the spatial distribution of the yearly mean PM_2.5_ concentration gradually shifted from the southern zone of Jianan District in Xuchang city to the northeast in 1998. However, by 2004, the center of gravity remained in the Jian'an District and then shifted to the northwest. The center of gravity was located at the junction of Yuzhou and Jian'an Districts in 2004 and has shown a trend of shifting to the northeast since then, it is correlated with the spatial distribution pattern showing a “northeast-southwest” movement in the PM_2.5_ concentration in Henan Province. The final yearly mean PM_2.5_ concentration centers of gravity in 2014 and 2019 were within Xinzheng, Zhengzhou city. Overall, the spatial center of gravity of the PM_2.5_ concentration in Henan Province showed a trend of drifting from due south to due north. This change can be attributed to the ongoing industrial development in Zhengzhou and the large base and fast growth rate of motor vehicle ownership, the increased pollution emissions from motor vehicles, and the industrial and energy structures in northern Henan. From the perspective of ellipse coverage, the area of the standard deviation ellipse first decreased year by year from 139,540,000 km^2^ in 1998 to 124,510,000 km^2^ in 2014, and then it increased to 125,380,000 km^2^ in 2019. This result indicates that its distribution range showed a “contraction-expansion” trend, which means that the main range of PM_2.5_ pollution expanded from 2014 to 2019. In addition, the standard deviation ellipse of 5 years covered the north, middle, and east of Henan Province, representing the vital area of air pollution reduction in Henan Province.

Overall, the PM_2.5_ pollution distribution in Henan Province has been expanding recently, a spatial spillover effect has emerged, and air pollution is developing toward regional pollution. These patterns are mainly related to the long-term economic development in some areas and the unreasonable industrial structure, followed by the Effects of terrain and meteorological environment. The wide range of airflow causes the local air quality to be affected by both local pollutant emissions and pollution sources from other areas.

### 3.3 Spatial aggregation analysis of PM_2.5_ concentration

#### 3.3.1 Global spatial autocorrelation analysis

**Figure 5:**
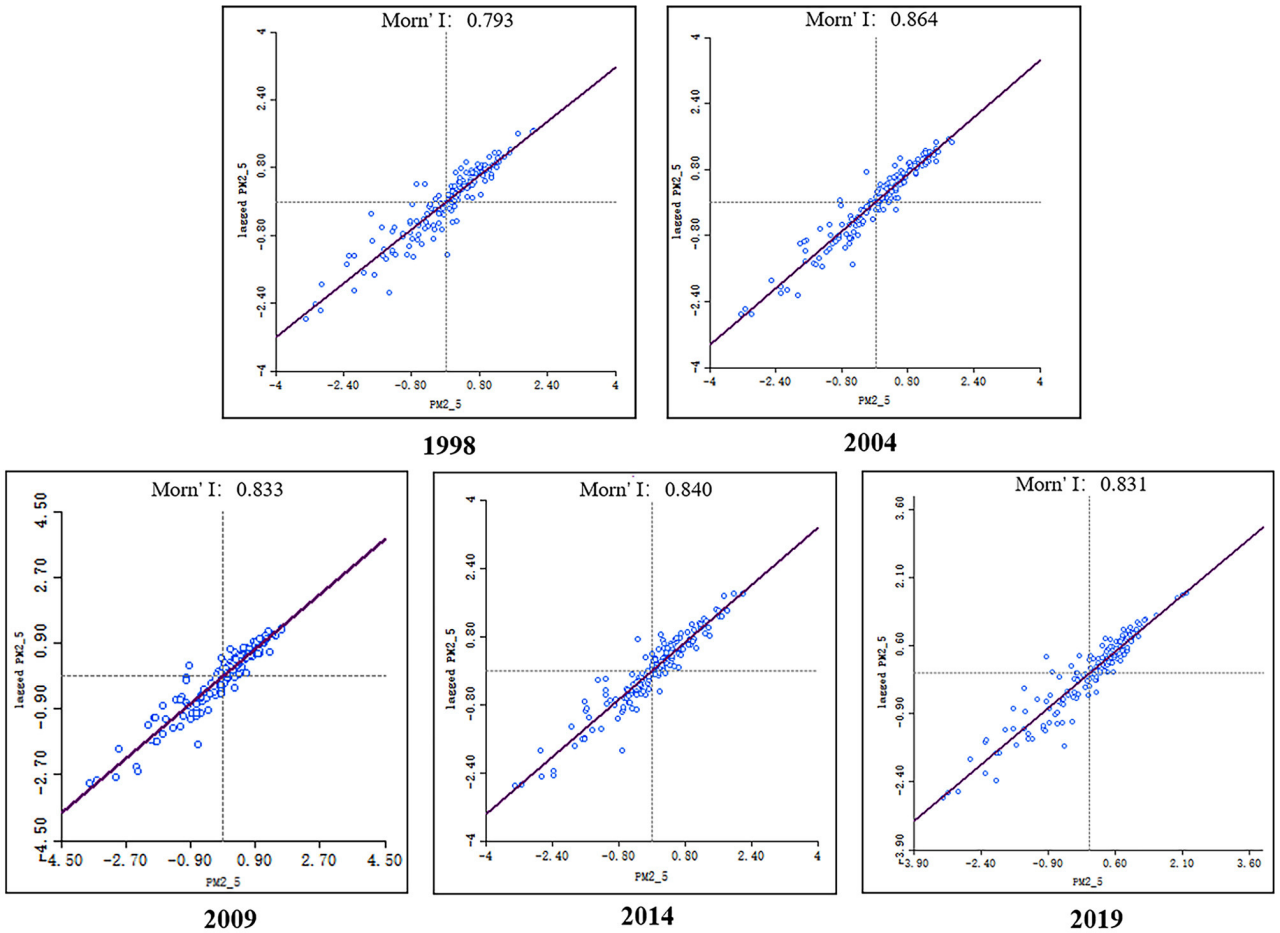
Moran's *I* index of PM_2.5_ for 1998, 2004, 2009, 2014, and 2019.

To analyze the spatial distribution characteristics of the PM_2.5_ concentrations in Henan Province, PM_2.5_ remote sensing inversion data at the county level of administrative units were used for zonal statistics, and the yearly mean PM_2.5_ data for 5 years, 1998, 2004, 2009, 2014 and 2019, were selected for the global spatial autocorrelation analysis of PM_2.5_ data in Henan Province. The Moran's I index was analyzed (Figure 5): In the selected 5 years, Moran's I index has been >0, showing that the spatial distribution of PM_2.5_ in Henan Province has a significant positive spatial correlation, i.e., a significant spatial aggregation characteristic. This finding also suggests that, while building the driving model, the spatial heterogeneity of the effects of numerous variables on PM_2.5_ should be taken into consideration.

#### 3.3.2 Local spatial autocorrelation analysis

**Figure 6:**
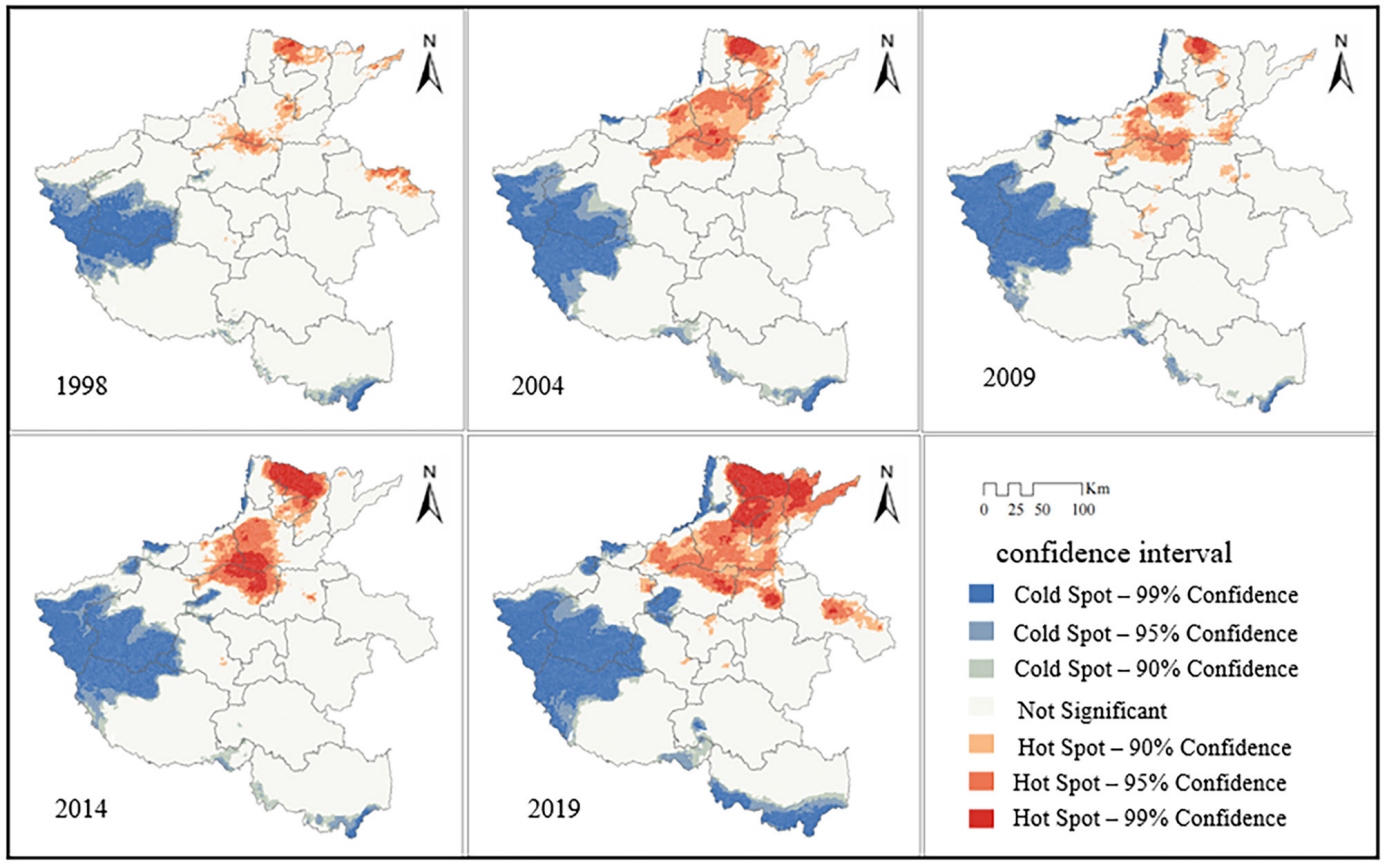
Spatial distribution of cold and hot spots.

In order to gain deeper insights into the spatial clustering attributes of PM_2.5_ in Henan Province, the hot spot analysis was performed using PM_2.5_ satellite inversion data, building upon the outcomes of global spatial autocorrelation analysis to investigate the local distribution pattern of PM_2.5_ concentrations. The results of the analysis (Figure 6) showed that the study area as a whole showed strong aggregation from 1998 to 2019, with local areas showing 99% confidence in the aggregation area in 1998, 2004, 2009, 2014, and 2019. From the perspective of spatial distribution, the hot spots are mainly dense in northern and central Henan, and the spatial correlation is high, and cold spots are dense in western and southern Henan, and the spatial correlation is low. The distribution of PM_2.5_ concentration cold and hot spots in Henan Province remained approximately the same, but the area and number of cold and hot spot areas of different levels changed to some extent. Temporally, the spatial autocorrelation of PM_2.5_ concentration in Henan Province indicated an overall strengthening trend, and the local autocorrelation in 2019 was significantly strengthened compared with that in 1998. Spatially, the hot spot areas showed a northward trend, gradually shifting from places such as Xuchang and Shangqiu to areas such as Anyang, Hebi, and Xinxiang in northern Henan.

### 3.4 Analysis of the driving mechanism

#### 3.4.1 Comparison of the GWR model and MGWR model

This study used 2019 PM_2.5_ concentration and driver data to construct the PM_2.5_ driver mechanism. In the results of the regression analysis, higher values of goodness-of-fit *R*^2^ and smaller values of AICc and RSS indicate a better and more accurate fit of the model. From the data in Table 4, it can be deduced that: the *R*^2^ in the MGWR is greater than that in the GWR model by 0.107; the AICc value for the MGWR is considerably smaller than that of the GWR model; in addition, the RSS of MGWR is smaller, which indicates that its predicted values are closer to the truth. As a result, this study's data analysis shows that the MGWR is more accurate than the classical GWR model and may be utilized to more precisely analyze the driving mechanisms of PM_2.5_ concentration.

**Table 4:** Comparison of GWR and MGWR models.

**Evaluation index**	**GWR**	**MGWR**
*R* ^2^	0.883	0.990
RSS	343.795	23.664
AICc	2,056.935	−4,667.184

**Figure 7:**
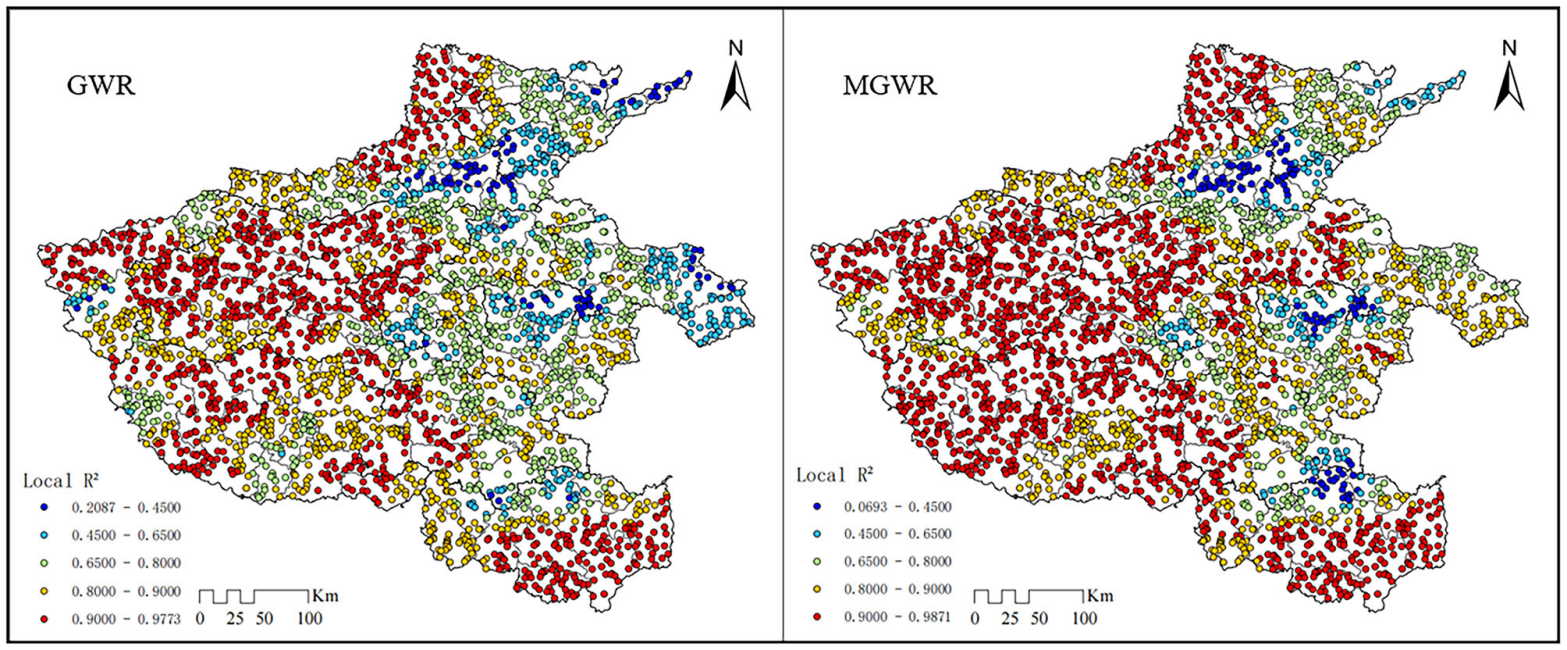
Comparison of local *R*^2^ spatial distribution between GWR and MGWR fitting results.

The *R*^2^ values in Figure 7 indicate the actual strength of the selected natural and socioeconomic indicators to explain the PM_2.5_ concentration levels. The local *R*^2^ of all samples ranged from 0.069 to 0.988. Among them, 77.80% of the sample points had an *R*^2^ of over 0.80, and 51.86% had an *R*^2^ of over 0.90. This indicates that the 12 selected influencing factors in this study demonstrate significant and comprehensive explanatory capability for the spatial distribution of PM_2.5_ concentration in Henan Province. The lower *R*^2^ values in some areas of Zhoukou, Xinxiang, and Zhumadian indicate the existence of possible missing factors and the possible influence of cross-border transports of air pollutants from neighboring provinces, resulting in a lower model fit in this area.

Based on the comparison of the bandwidth between the GWR model and the MGWR model (Table 5), it is evident that the MGWR model can explicitly represent the varying effect scales of different variable quantities, whereas the GWR can only represent the average effect scales of each variable. The bandwidth of the variables measures the spatial scales of action of each process, which can reflect the differences in the scales of action of different natural and socioeconomic influences on PM_2.5_ concentrations. The larger the action scale is, the smaller the spatial heterogeneity of the effect of the driving factor, while the opposite spatial heterogeneity is larger.

**Table 5:** Comparison of bandwidth between the GWR model and MGWR model.

**Factor**	**Bandwidth of GWR**	**Bandwidth of MGWR**
Intercept	86	43
X1: surface temperature	86	182
X2: relative humidity	86	1,194
X3: wind speed	86	225
X4: precipitation	86	43
X5: vegetation index	86	1,101
X6: elevation	86	43
X7: GDP	86	46
X8: nighttime light	86	686
X9: CO_2_ emissions	86	52
X10: electricity consumption	86	1,843
X11: population density	86	2,955
X12: arable land area share	86	44

Table 5 shows that the MGWR provides a more detailed perspective by directly reflecting the differential effect scales of various variables, while the GWR only considers the average effect scale. In addition, the bandwidth of variables is used to measure the spatial scale of their effects, revealing the differences in the scale of natural and socio-economic impacts on PM_2.5_ concentration. The larger the scale of action, the smaller the spatial heterogeneity of influencing factor effects. The smaller the scale of action, the greater the spatial heterogeneity.

Among the natural factors, the precipitation, elevation, mean surface temperature, and mean wind speed were 43, 43, 182, and 225, respectively, which were small (<300), and their effects on PM_2.5_ concentration were more spatially heterogeneous. The effect scales of GDP, the proportion of cultivated land, and CO_2_ emissions in the socioeconomic factors were 46, 44, and 56, respectively, all of which were relatively small (<100). Their effects on PM_2.5_ concentrations showed significant spatial heterogeneity. The effect scale of nighttime lights was 686, and its influence on PM_2.5_ concentration exhibited certain spatial heterogeneity. The effect scale of electricity consumption was 1,843, the spatial heterogeneity was relatively small. The effect scale of population density was 2,955, which was equal to the total sample size and was a global variable. There was almost no spatial heterogeneity, and the impression on PM_2.5_ concentration was consistent throughout Henan Province.

#### 3.4.2 Spatial pattern of regression coefficient coefficients of drivers

Table 6 shows the statistical results of the regression coefficients of the MGWR model, and the results show the estimated coefficients of the MGWR using standardized data and the proportion of each influence factor coefficient for the different directions of PM_2.5_ action. The regression coefficients of the influencing factors derived from the MGWR model indicate that there is spatial heterogeneity in the effects of each indicator on regional PM_2.5_ concentrations. where surface temperature and humidity have positive effects; additionally, GDP, night lighting, and CO_2_ emissions mainly have positive effects, accounting for 93.70, 81.24, and 98.40% of the total sample, respectively. There is both a positive and a negative effect on the proportion of cultivated land area, with spatial polarization, in which the positive effect accounts for 57.37% of the total sample and the negative effect accounts for 47.64% of the total sample. Wind speed and NDVI were negative, and precipitation and elevation were mainly negative, accounting for 90.60 and 98.54% of the total samples, respectively. The intensity of the above drivers on PM_2.5_ concentrations, in order from highest to lowest, as seen through the absolute values of the coefficient means, are as follows: CO_2_ emissions (3.452), elevation (0.353), GDP (0.285), precipitation (0.271), intercept (0.231), surface temperature (0.054), wind speed (0.022), arable land area share (0.009), relative humidity (0.006), nighttime light (0.006), electricity consumption (0.002), vegetation index (0.001), and population density (0.001).

**Table 6:** Parameter estimates for the regression of PM_2.5_ concentrations using MGWR.

**Variables**	**MGWR coefficients**			**Percentage of cities by significance (95% level) of** ***t test***		
	**Mean**	**Min**	**Max**	***P* ≤ 0.05 (%0)**	**+(%)**	**–(%)**
Intercept	0.231	−1.027	0.990	94.55	73.17	26.83
X1	0.054	−0.026	0.175	60.59	100.00	0.00
X2	0.006	−0.008	0.024	34.17	100.00	0.00
X3	−0.022	−0.114	0.021	29.74	0.00	100.00
X4	−0.271	−1.320	0.372	77.03	9.40	90.60
X5	−0.001	−0.015	0.011	13.94	0.00	100.00
X6	−0.353	−1.173	0.258	92.83	1.46	98.54
X7	0.285	−0.607	1.338	45.70	93.70	6.30
X8	0.006	−0.024	0.033	31.56	81.24	18.76
X9	3.452	−2.400	13.189	57.24	98.40	1.60
X10	−0.002	−0.009	0.002	0.00	0.00	0.00
X11	−0.001	−0.003	0.000	0.00	0.00	0.00
X12	−0.009	−0.442	0.529	57.37	52.36	47.64

**Figure 8:**
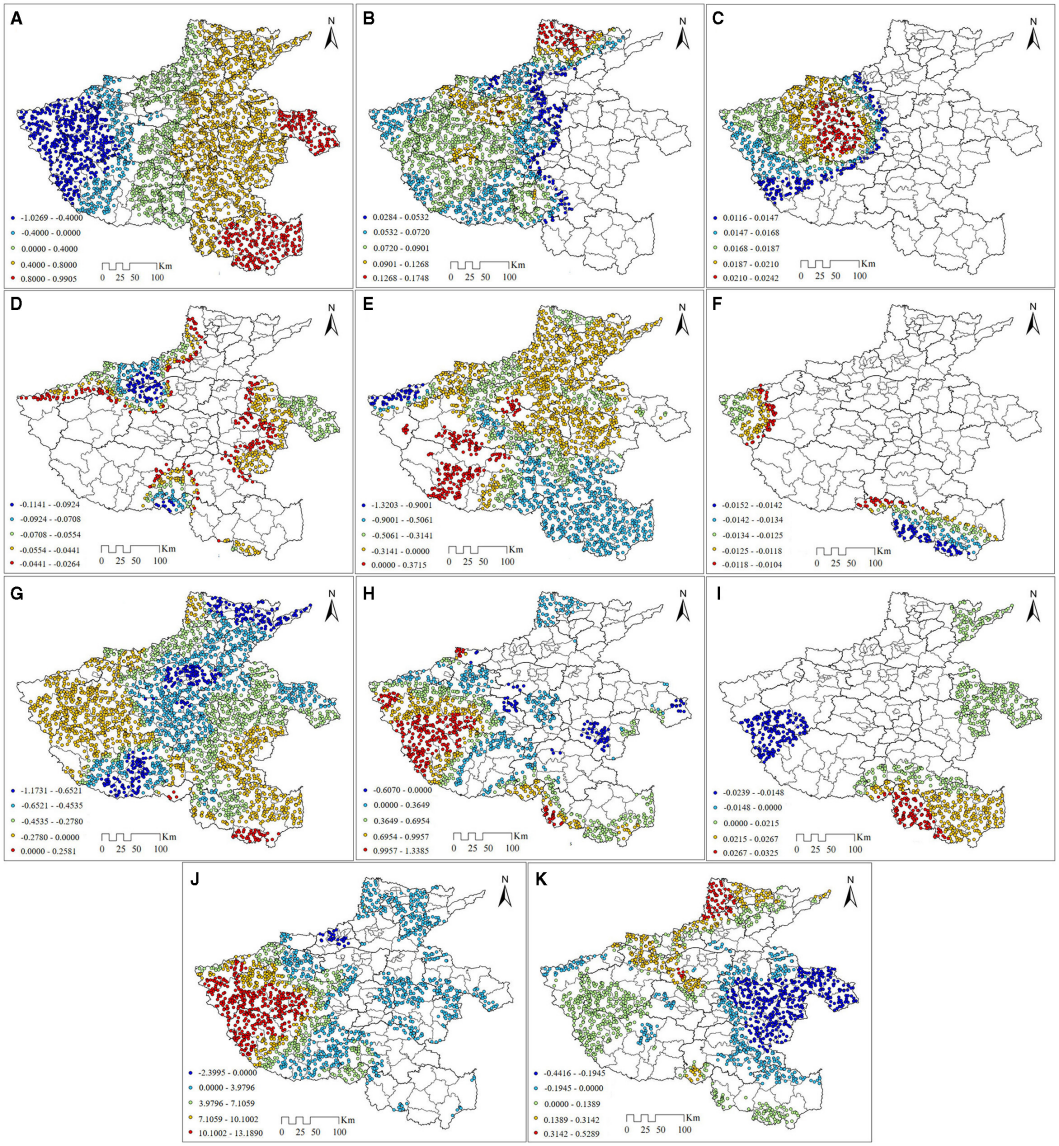
Spatial pattern of MGWR coefficients. **(A)** Intercet (bandwith = 43). **(B)** X1 (bandwith = 182). **(C)** X2 (bandwith = 1194). **(D)** X3 (bandwith = 225). **(E)** X4 (bandwith = 43). **(F)** X5 (bandwith = 1101). **(G)** X6 (bandwith = 43). **(H)** X7 (bandwith = 46). **(I)** X8 (bandwith = 686). **(J)** X9 (bandwith = 52). **(K)** X12 (bandwith = 44).

As seen in Figure 8, In the western part of Henan Province, the intercept parameter estimates are significantly negative, while in the rest of the area, the parameter estimates are significantly positive. This suggests that the existence of covariates has some influence on the distribution of PM_2.5_ concentrations in Henan Province. The lower PM_2.5_ concentration in western Henan may be related to the complex topography and climate, while the spread and dispersion of PM_2.5_ in the rest of the plain-dominated areas may be relatively easy (Figure 8A). The surface temperature has a positive influence on the PM_2.5_ concentration within a significant range, and the effect is strongest in the Henan section of China's second- and third-level step junctions. This result is because the fact that the junction of the second and third-level steps is in a basin surrounded by mountains. This topographic obstruction plays an important role in aerosol accumulation to some extent (56) (Figure 8B). The influence of relative humidity on PM_2.5_ concentration shows a positive effect, and the significant area has a circular structure, with the bordering areas of Zhengzhou, Luoyang, and Pingdingshan cities at the center; the center of gravity of influence intensity decreases to the outer side, which may be caused by the more prominent local altitude. Foggy weather with high relative humidity will lead to the inverse temperature phenomenon near the ground, which is not conducive to the diffusion of particulate matter (57), making the combination of particulate matter and water mist form PM_2.5_. However, when the relative humidity exceeds a certain threshold value, it causes precipitation, and the precipitation will have a flushing effect on the air to reduce the PM_2.5_ concentration. Since the relative humidity in Henan Province is below the threshold value, its effect on reducing PM_2.5_ concentration is positive (Figure 8C). Wind speed negatively affects the PM_2.5_ concentration, which indicates that airflow movement can effectively reduce the local PM_2.5_ concentration under windy conditions with good air pollutant dispersion conditions (Figure 8D). Precipitation mainly has a suppressing effect on PM_2.5_, with positive promotion effects found only in the southern part of Luoyang city and the central part of Nanyang city. In the rest of Henan Province, precipitation shows a significant northwest-southeast directional effect. Xinyang city, Zhumadian city, Pingdingshan city, and Sanmenxia city are high-value areas, and the influence intensity decreases on both sides (Figure 8E). The NDVI has an suppressed effect on PM_2.5_ concentration in the significant area, and when the NDVI value is high, it indicates that vegetation has a stronger absorption and deposition effect on atmospheric particulate matter, thus contributing to the diminution of PM_2.5_ concentration (Figure 8F). Elevation has a significant suppressed influence on the PM_2.5_ concentration in most parts of the province and has a certain positive promoting influence in only a small area south of Xinyang city. The inhibition effect forms the highest intensity in the northeast-southwest direction in Henan Province and decreases to the two sides, which is consistent with the topographic features of Henan Province (Figure 8G). In the majority of regions within Henan Province, GDP and PM_2.5_ concentrations show a positive correlation, which is due to the increase in emissions from pollution sources brought about by industrial development closely related to GDP, and only in some areas of Zhoukou city, Zhengzhou city and Xuchang city, it inhibits PM_2.5_, which is explained by the local government's increased investment in air control funds (Figure 8H). The negative effect of nighttime lighting on PM_2.5_ is mainly concentrated in Luoyang and Nanyang. Nighttime lighting can, to a certain extent, reflect the local economic development level (58). The negative impact of nighttime lighting on PM_2.5_ in these areas may be due to the tendency of Luoyang and Nanyang, which have a relatively developed level of economic development, to implement more stringent environmental protection measures (Figure 8I). There is a noticeable correlation between CO_2_ emissions and PM_2.5_ levels in the majority of areas in Henan Province, and the influence of CO_2_ emissions on PM_2.5_ increases from the northeast to the southwest. The explanation for this is that the primary origin of carbon dioxide emissions lies in the combustion of fossil fuels like coal, oil, and gas during industrial production (59). Consequently, the gases and particulate matter discharged due to industrial development play a crucial role in the rise of PM_2.5_ concentrations (Figure 8J). The arable land area share has strong spatial heterogeneity, and there is a bipolar influence on the PM_2.5_ concentration, with the capacity to suppress the PM_2.5_ in the plain areas of the province due to the larger and more aggregated area of cultivated land in the plain areas, and the plants in farmland have an adsorption influence on PM_2.5_, this is consistent with Yang et al. (46) findings that cropland has a negative effect on PM_2.5_ concentration in most areas of Zhengzhou City. The positive impact of cropland proportion on PM_2.5_ concentrations in mountainous and hilly regions may be attributed to the fact that an increase in cropland proportion leads to a decrease in forested land, resulting in an amplified extent of bare land during winter, thereby augmenting the PM_2.5_ source (60) (Figure 8K).

## 4 Conclusion and discussion

Lately, there has been considerable focus on the spillover of PM_2.5_ on air quality and public health. Taking Henan Province as the research object, this study used nearly 22 years of satellite remote sensing data from 1998 to 2019 to study the spatiotemporal distribution properties of PM_2.5_ in this region and deeply understand the influence of various driving factors on PM_2.5_ concentration and its spatial heterogeneity. The analysis presented above led this text to the following conclusions:

(1) PM_2.5_ management in Henan Province has achieved certain results in recent years, but there are still most districts and counties where the annual average concentration of PM_2.5_ exceeds the national category 2 standard, which indicates that there is still a long way to go in managing PM_2.5_ pollution in Henan Province.
(2) Between 1998 and 2019, the center of gravity of PM_2.5_ concentration in Henan Province as a whole shifted to the north, and the northern part of Henan Province is the key area for air pollution prevention and control.
(3) The PM_2.5_ concentrations from 1998 to 2019 have obvious spatial autocorrelation and significant spatial aggregation characteristics. Constructing the PM_2.5_ driving mechanism based on the MGWR model can reveal the effect of each influencing factor on different spatial scales, and a more realistic fitting effect can be derived. The comparison of the two models showed that the MGWR model was more applicable to the study of the PM_2.5_ concentration driving mechanism.
(4) Based on revealing the strength of each influencing factor, the regression coefficients of the influencing factors derived from the MGWR model in this study indicated that there were different degrees of spatial heterogeneity in the influence of each factor on PM_2.5_ concentrations in Henan Province.

From 1998 to 2019, the PM_2.5_ levels in Henan Province exhibited an “M”-shaped trend, with an overall increase in PM_2.5_ concentration in 2019 compared to 1998. The spatial center of gravity shift of the PM_2.5_ concentration in Henan Province from 1998 to 2019 showed an overall northward trend, with an overall “S” shape. The spatial center of gravity of the PM_2.5_ concentration was in within Zhengzhou city in 2019. This result was related to the continuous development of industry in Zhengzhou city, the large base and rapid growth rate of motor vehicle ownership, and the increased pollution emissions from motor vehicles. During the whole time interval, the overall PM_2.5_ center of gravity shift in Henan Province showed a northward trend, which was in line with the trend of the PM_2.5_ center of gravity shift in Henan Province by Ge QX (27). On this basis, we categorized the annual average PM_2.5_ concentrations in all districts and counties of Henan Province according to the Ambient Air Quality Standards (GB3095-2012) and found that overall, in Henan Province, although the PM_2.5_ pollution situation has been alleviated, most districts and counties still do not meet the national Class II standards for PM_2.5_ concentrations.

The PM_2.5_ concentrations from 1998 to 2019 had obvious spatial autocorrelation and significant spatial aggregation characteristics. The local spatial correlation analysis showed that the spatial hot spot areas were mainly dense in northern Henan Province, containing Anyang city, Hebi city, Xinxiang city, and Jiaozuo city, which coincided with the industrial structure and energy structure in northern Henan Province. Therefore, from a sustainable point of view, industrial upgrading and transformation should be encouraged or made mandatory, and there should be a shift from traditional industries that are highly polluting and emit high levels of pollutants to new industries that are clean, efficient, and emit low levels of pollutants. The cold spot areas were mainly dense in the west and south of Henan Province, including Sanmenxia city, Nanyang city, etc. This result was related to the topography and landscape, as western and southern Henan have many mountain ranges, which play the role of isolating PM_2.5_ transmission, so western and southern Henan have weaker PM_2.5_ aggregation than other areas in Henan Province.

The results of the MGWR model show that the effects of most of the influencing factors on PM_2.5_ concentration are spatially heterogeneous, which is consistent with the results of the Zang et al. (26) study based on the GWR model. Different from that, we reveal the different scales of the role between PM_2.5_ concentration and each influencing factor in Henan Province based on the MGWR model. We found that the bandwidths of precipitation, elevation, GDP, cropland ratio, and CO_2_ emission are smaller, which indicates that their effects on PM_2.5_ concentration are more spatially heterogeneous, and the multiscale effects should be taken into account when PM_2.5_ management or analysis is carried out through these factors.

Influencing factors such as CO_2_ emissions, elevation, GDP, and precipitation had a strong influence on PM_2.5_ concentration, which was consistent with the findings of Zang et al. (26) and Wu et al. (61). Unlike that, our results point out the spatial heterogeneity of the effects of most of the influencing factors on PM_2.5_ concentrations and reveal the effects of the influencing factors at the optimal scale. It is noteworthy that the proportion of arable land presents a differential effect on PM_2.5_ concentration in Henan Province, which is in line with the results of Hong K-r's study (62). Cultivated land, like common vegetation, can effectively reduce PM_2.5_, but the effect of cultivated land on PM_2.5_ mainly depends on the intensity of agricultural activities, so the scientific utilization of cultivated land and the adjustment of its function have a positive effect on the management of PM_2.5_.

The current study mainly focuses on analyzing the multi-scale effects of the role of PM_2.5_ influencing factors in Henan Province. In the next study, we plan to expand the study area to the Yellow River basin to form regional research results. In addition, considering that the results of the study show the existence of covariate effects, we plan to introduce more factors that may have an effect on the PM_2.5_ concentration and to study in depth the effects of the factors on PM_2.5_ at different scales, so as to provide more valuable references for the prevention and control of air pollution in China.

## Data availability statement

The original contributions presented in the study are included in the article/supplementary material, further inquiries can be directed to the corresponding author/s.

## Author contributions

HW: Conceptualization, Funding acquisition, Project administration, Resources, Supervision, Writing – review & editing. MZ: Formal analysis, Investigation, Methodology, Visualization, Writing – original draft. JN: Funding acquisition, Resources, Supervision, Validation, Writing – review & editing. XZ: Formal analysis, Funding acquisition, Validation, Writing – review & editing.
